# Prevalence and sociodemographic characteristics of parents among patients treated in adult psychiatric hospitals – a register-based nationwide study

**DOI:** 10.3389/fpsyt.2025.1654760

**Published:** 2025-09-03

**Authors:** Signe Heuckendorff, Rene B. K. Brund, Julie Buhl-Wiggers, Anne A. E. Thorup, Anne Ranning, Anne Dorothee Müller, Kirsten Fonager

**Affiliations:** ^1^ Psychiatry, Aalborg University Hospital, Aalborg, Denmark; ^2^ Department of Social Medicine, Aalborg University Hospital, Aalborg, Denmark; ^3^ Danish Mental Health Foundation, Copenhagen, Denmark; ^4^ Department of Economics, Copenhagen Business School, Frederiksberg, Denmark; ^5^ Department of Clinical Medicine, University of Copenhagen, Copenhagen, Denmark; ^6^ Child and Adolescent Mental Health Center, Copenhagen University Hospital – Mental Health Services Copenhagen, Copenhagen, Denmark; ^7^ Copenhagen Research Center for Mental Health, Mental Health Centre Copenhagen, Copenhagen, Denmark; ^8^ Department of Psychology, University of Copenhagen, Copenhagen, Denmark; ^9^ Department of Clinical Medicine, Aalborg University, Gistrup, Denmark

**Keywords:** parental mental illness, childhood adversity, mental health services, family interventions, prevalence, health policy

## Abstract

**Introduction:**

Children of parents with mental illness face heightened risks of adverse health, social, and educational outcomes. Yet, families affected by parental mental illness are often overlooked in mental health and social care systems. Identifying parents in psychiatric care enables targeted interventions to mitigate intergenerational risks. This study aims to estimate the prevalence and describe the sociodemographic characteristics of patients in psychiatric hospital services in Denmark who are parents, with particular focus on those living with minor children, to inform future preventive support strategies.

**Methods:**

Using Danish nationwide register data from 2020-2022, we identified individuals with at least one adult psychiatric hospital contact. Parenthood was determined through linkage to the Civil Registration System, including children aged 0–29 years. Diagnoses, socioeconomic status, and living arrangements were described, and associations between parental characteristics and co-residence with a minor child were described using crude relative risks.

**Results:**

Among 164,223 patients in psychiatric hospital services, 24% had at least one minor child (aged 0–17 years), and 8% had only adult children (aged 18–29 years). The most common diagnoses among parents with minor children were neurotic and stress-related disorders (31%) and affective disorders (25%). Geographic and diagnostic variation in the prevalence of parents was observed, and parent patients generally had higher socioeconomic status than non-parent patients. Among parent patients, those with a partner, higher education, and labor market affiliation were more likely to live with their child.

**Conclusions:**

One in four Danish psychiatric hospital patients have minor children, highlighting the need for systematic identification and tailored services. The findings underscore the importance of integrating family-focused approaches within psychiatric care to reduce the long-term burden on children and break cycles of mental illness.

## 1 Introduction

Children of parents with mental illness face a wide range of adverse health and social risks. These include poorer mental health and early onset of mental disorders (1–4), poorer physical health (5, 6), negative social outcomes such as lower graduation rates (7) and higher likelihood of conviction for crime (8). Further, parental mental illness may impact family dynamics and access to social resources leading to fewer protective or buffering factors than in families without parental mental illness (9).

In Denmark, research has shown that approximately two in five children aged 0–16 years have parents with a mental health condition, and 13% have parents who received treatment in adult psychiatric hospital service (10). Despite this high prevalence, these children are often overlooked in both mental health care systems and in social systems in the surrounding society. While some parents with mental illness experience periods of remission or recovery and thus can provide a stable and supportive home environment, many still face challenges that can affect their ability to care for their children, particularly during episodes of symptoms and active illness. Identifying families in need of support provides a window of opportunity for early intervention with tremendous preventive potential for these children.

Psychiatric hospitals (i.e., both wards and outpatient clinics) present a key opportunity for the detection of children in need of support while their parents are in treatment. A systematic, top-management prioritized approach is essential for effective identification and subsequent support. Moreover, it is acknowledged that the social environment is critical to patient recovery and thus knowing more about the importance of parenting for the patient, and the impact of family dynamics for parents as patients in psychiatry is of most importance. Few studies have investigated the effect of involving the family in the treatment of psychiatric disorders. However, Reupert et al. found that the role of being a parent is an important motivation for the recovery of parents in psychiatric care, that is often overlooked in clinical measures (11).

Still, data on the prevalence of parents among patients in psychiatric hospital services is lacking but needed to inform clinicians, service providers, and decision-makers. Accurate estimates support the development of targeted interventions, improve clinical identification and guide resource allocation. However, many countries lack comprehensive data, limiting researchers’ ability to provide evidence-based recommendations. Addressing this gap by analyzing prevalence rates and associated risk and resilience factors is crucial for strengthening mental health services and ensuring appropriate support for affected families (12).

One way to describe the prevalence of parental mental illness is a population-level “top-down” approach, where the prevalence of children living with parents who have a mental illness is estimated (12). Population-based cohort studies using this approach have reported that 12.8% of children in Denmark (10) and 11% in Sweden (13) live with a parent receiving hospital-based psychiatric care. However, such population-level prevalence is not useful in clinical everyday life. Another approach to estimating prevalence is the “bottom-up” approach that determines the number of patients in the psychiatric settings who are parents (12). This method provides a prevalence that is helpful in a clinical context.

While studies from Australia, Norway, and Sweden provide some prevalence data, research on this issue in Denmark remains limited. In Australia, a study across New South Wales reported that 25-28% of current patients in psychiatric hospital services were parents, based on annual one-day census data from 2008-2011 (14). A large Australian study spanning 2003–2011 showed that approximately 20% of patients in psychiatric hospital services were parents (15), and a Czech study reported a prevalence of 35% of patients with severe mental illness were parents, including those with adult offspring (16). A Swedish study from 2001 reported that 36% of adult patients in psychiatric hospital services had children under 18 years of age (17), while a Norwegian study from 2013 found a 36% prevalence among psychiatric outpatients (18).

However, audit-based studies such as these (14, 17, 18) are often restricted to specific regions and modest sample sizes, limiting their generalizability. Additionally, patients with mental illness may avoid disclosing their parenting status in mental health services due to fear of child protection involvement (18), while also concealing their mental health challenges in social services to avoid stigmatization. This dual concern can lead to under-identification and inadequate support. Further, data collection is resource-intensive, which may contribute to incomplete and unrepresentative findings.

The provision of preventive family interventions including psychoeducation with parents within adult mental health settings has demonstrated positive outcomes for children (19–21) as well as improving family psychosocial functioning (21). Thus, effective interventions do exist, but prior identification is needed to be able to intervene. A review highlights the importance of early detection and preventive interventions in clinical settings. It recommends that mental health professionals place greater emphasis on prevention and early identification in daily practice to reduce the burden on children as relatives, and over time, to lower the incidence of mental illness or change expected trajectories to less debilitating outcomes (22). Unlike Norway and Sweden, where laws are made to ensure focus on children as relatives, Denmark lacks regulations mandating the identification of children whose parents suffer from a mental illness. While the Danish Child’s Law requires all public professionals, including mental health staff, to report any concerns regarding children’s welfare (23), interventions and guidelines for how to serve this vulnerable group are inconsistent across and within Danish regions (24). Despite recommendations from the Danish Health Authority to prioritize children of parents with a mental illness (25), a systematic, nationwide effort has yet to be implemented.

To address the knowledge gap, this study aims to estimate the prevalence of parenthood among patients treated in Danish psychiatric hospitals. Specifically, we focus on patients with minor children and describe their sociodemographic characteristics, including partnership status, education, with particular attention to those living with their children. Additionally, we explore geographic variation in the prevalence of parenthood across regions and municipalities to inform future, locally tailored, preventive and family-focused support strategies.

## 2 Materials and methods

### 2.1 Study design and settings

This study utilized the Danish nationwide registers. Denmark’s tax-funded healthcare system provides free access to healthcare, including mental health services, with a few private outpatient clinics also in operation (26). The healthcare administration is managed by five regions, each with varying population sizes. The psychiatry specialty is organized separately from other medical specialties. Responsibility for social care and related interventions falls to the 98 municipalities.

The regional mental health services are structured into two main sectors: the primary healthcare sector, which include general practitioners (GPs) and practicing psychiatrists, and the secondary healthcare sector, which encompasses psychiatric hospitals and related outpatient services. It is estimated that GPs manage 80–90% of minor psychiatric disorders, such as mild to moderate depression and non-comorbid anxiety conditions. GPs also serve as ‘gatekeepers’, referring patients to either practicing psychiatrists or to treatment within the hospital sector, and they perform medical controls (26). The secondary sector provides a range of services, including inpatient and outpatient facilities, as well as community psychiatry, with variations between regions likely influenced by demographic differences, prioritization, and funding.

Addiction services such as treatment of alcohol or drug addiction are organized and provided by the municipalities. We were not able to include information on parents in treatment for addiction.

### 2.2 Data sources

From the Danish Civil Registration System (27) information on the individuals’ children, age, sex, geography, and cohabitation were obtained.

Information from the psychiatric hospital services were provided from the National Patient Register (28) and included information on inpatient and outpatient contacts and diagnoses that used the International Classification of Diseases, Tenth Revision (ICD-10) codes.

The parents’ highest level of completed education was extracted from the Population Education Registry (29), and The Income Statistics Register provided information on socioeconomic classification (30).

Data linkages were achieved via the personal identity number that is assigned to all Danish residents at birth or when individuals become residents. The register keepers at Statistics Denmark carried out the data collection and register linkage. All the data were deidentified before the researchers received access.

### 2.3 Study population

We included all individuals who had contact with adult psychiatric hospitals and related outpatient services between January 1st, 2020, and December 31st, 2022. Their children were identified if they were aged 0–29 years in the study period. Individuals with contact to the geriatric psychiatry or child- and adolescent psychiatry were excluded from the study.

### 2.4 Variables

All variables were measured in the calendar year of the patient’s first contact with psychiatric hospital services during the study period (2020–2022), except ‘living with child’, geographical region and municipality that was based on residence of the patient in the beginning of the study period.

Based on the primary ICD-10 diagnosis from the patient record, the diagnoses were grouped according to the main DF-category, see **Box 1**.

Box 1 **ICD-10 diagnostic categories used in the study.**

**Table:** 

ICD-10 code	ICD-10 diagnosis
DF00-DF09	Organic, including symptomatic, mental disorders
DF10-DF19	Mental and behavioral disorders due to psychoactive substance use
DF20-DF29	Schizophrenia, schizotypal and delusional disorders
DF30-DF39	Mood [affective] disorders
DF40-DF48	Neurotic, stress-related and somatoform disorders
DF50-DF59	Behavioral syndromes associated with physiological disturbances and physical factors
DF60-DF69	Disorders of adult personality and behavior
DF70-DF79	Intellectual disability
DF80-DF89	Disorders of psychological development
DF90-DF98	Behavioral and emotional disorders with onset usually occurring in childhood and adolescence
DF99	Unspecified mental disorder
DZ00-DZ99	Factors influencing health status and contact with health services

In cases of patients with more than one primary diagnosis during the three years, the earliest diagnosis in the ICD-hierarchy, was included. Patients were categorized as outpatients if they had solely outpatient contacts during the study period. If they had at least one inpatient contact, they were categorized as inpatients.

The variable ‘lives with child’ describes whether the patient shared the same address as the child. For patients with both minor and adult children, cohabitation status was determined based solely on living with af minor child. These patients were classified as having minor children. If the child partly resides with but does not have the same address as the parent who is a patient, it is categorized as not living with child.

Educational status was based on the levels of the International Standard Classification of Education (ISCED 2011) (31) and grouped into three categories according to the parent’s highest educational attainment. These were level 0–2 if they had 10 years of mandatory education or below, level 3–4 for 10–14 years of education, such as high school, and level 5–8 for more than 14 years of education, such as a bachelor’s degree. Socioeconomic position was dichotomized into “affiliated” and “not affiliated” with labor marked or education. Individuals were classified as affiliated if they were employed, enrolled in education, or receiving temporary benefits such as unemployment or sickness benefits. Individuals receiving disability pension, cash benefits, or other forms of long-term public support were classified as not affiliated (30).

### 2.5 Data analyses

We calculated the prevalence of patients in adult psychiatric hospital services who had at least one child aged 0–29 years, and presented descriptive sociodemographic characteristics stratified by parental status. Separate prevalence estimates were provided for patients with minor children (aged 0–17 years) and patients with only adult children (aged 18–29 years). Patients with both minor and adult children were classified under the minor children group.

To describe the association between parental characteristics and co-residence with minor children, we estimated relative risks (RRs) and 95% confidence intervals. This analysis was restricted to patients identified as parents of at least one minor child, comparing those who were living with their child to those who were not. RRs were calculated as simple ratios of proportions within each characteristic group (e.g., females vs. males). We did not adjust for covariates in these analyses since the aim of the study solely was descriptive.

## 3 Results

We identified 164,223 individuals in total with at least one contact with the adult psychiatric hospitals and related outpatient services between 2020-2022 (**Table 1**). Of these, 24% had at least one child aged 0–17 years (minor children) and 8% had only child(ren) aged 18–29 years. In the following, we focus on the group of patients with minor children.

**Table 1:** Baseline characteristics.

	Overall population	Patients with at least minor child	Patients with solely child(ren) aged 18-29 years	Patients with no children
	N=164,223	N=39,153	N=13,859	N=111,211
Prevalence of parenthood		24%	8%	
Age				
Mean (SD)	39.9 (17.3)	39.1 (8.48)	54.2 (6.18)	38.4 (19.6)
Median [Min, Max]	35.5 [18.0, 105]	38.7 [18.0, 79.8]	54.2 [34.7, 88.5]	29.7 [18.0, 105]
Sex				
Male	73087 (44.5%)	15790 (40.3%)	5597 (40.4%)	51700 (46.5%)
Female	91136 (55.5%)	23363 (59.7%)	8262 (59.6%)	59511 (53.5%)
Type of contact				
Outpatient	72723 (44.3%)	17544 (44.8%)	5347 (38.6%)	49832 (44.8%)
Inpatient	91500 (55.7%)	21609 (55.2%)	8512 (61.4%)	61379 (55.2%)
Diagnosis				
DF00–09 Organic, including symptomatic, mental disorders	7305 (4.4%)	339 (0.9%)	495 (3.6%)	6471 (5.8%)
DF10–19 Mental and behavioral disorders due to psychoactive substance use	13075 (8.0%)	2777 (7.1%)	1659 (12.0%)	8639 (7.8%)
DF20–29 Schizophrenia, schizotypal and delusional disorders	28630 (17.4%)	3622 (9.3%)	1742 (12.6%)	23266 (20.9%)
DF30–39 Mood [affective] disorders	36149 (22.0%)	9648 (24.6%)	4124 (29.8%)	22377 (20.1%)
DF40–48 Neurotic, stress-related and somatoform disorders	36140 (22.0%)	12301 (31.4%)	3517 (25.4%)	20322 (18.3%)
DF50–59 Behavioral syndromes associated with physiological disturbances and physical factors	4187 (2.5%)	936 (2.4%)	240 (1.7%)	3011 (2.7%)
DF60–69 Disorders of adult personality and behavior	9917 (6.0%)	3109 (7.9%)	503 (3.6%)	6305 (5.7%)
DF70–79 Intellectual disability	2779 (1.7%)	77 (0.2%)	11 (0.1%)	2691 (2.4%)
DF80–89 Disorders of psychological development	2881 (1.8%)	258 (0.7%)	35 (0.3%)	2588 (2.3%)
DF90–98 Behavioral and emotional disorders with onset usually occurring in childhood and adolescence	6250 (3.8%)	1928 (4.9%)	185 (1.3%)	4137 (3.7%)
DF99 Unspecified mental disorder	1107 (0.7%)	259 (0.7%)	60 (0.4%)	788 (0.7%)
DZ00–99 Factors influencing health status and contact with health services	15803 (9.6%)	3899 (10.0%)	1288 (9.3%)	10616 (9.5%)
Living with the child				
No	139408 (84.9%)	16510 (42.2%)	11687 (84.3%)	111211 (100%)
Yes	24815 (15.1%)	22643 (57.8%)	2172 (15.7%)	0 (0%)
Region				
North	13934 (8.5%)	3355 (8.6%)	1144 (8.3%)	9435 (8.5%)
Central	33256 (20.3%)	7647 (19.5%)	2563 (18.5%)	23046 (20.7%)
Southern	40797 (24.8%)	10973 (28.0%)	3824 (27.6%)	26000 (23.4%)
Capital	53724 (32.7%)	11855 (30.3%)	4229 (30.5%)	37640 (33.8%)
Zealand	22512 (13.7%)	5323 (13.6%)	2099 (15.1%)	15090 (13.6%)
Educational status				
<10 years (ISCED 0-2)	68695 (41.8%)	13173 (33.6%)	4414 (31.8%)	51108 (46.0%)
10–14 years (ISCED 3-4)	58371 (35.5%)	13968 (35.7%)	5700 (41.1%)	38703 (34.8%)
>14 years (ISCED 5-8)	32295 (19.7%)	11080 (28.3%)	3297 (23.8%)	17918 (16.1%)
Missing	4862 (3.0%)	932 (2.4%)	448 (3.2%)	3482 (3.1%)
Number of unique children				
n	106458	82597	23861	0
Socioeconomic position				
Affiliated to labor marked or education	84216 (51.3%)	21653 (55.3%)	6305 (45.5%)	56258 (50.6%)
No affiliation to labor marked or education	57585 (35.1%)	12099 (30.9%)	6369 (46.0%)	39117 (35.2%)
Missing	22422 (13.7%)	5401 (13.8%)	1185 (8.6%)	15836 (14.2%)
Living with partner				
No	101003 (61.5%)	16899 (43.2%)	7848 (56.6%)	76256 (68.6%)
Yes	58154 (35.4%)	21533 (55.0%)	5749 (41.5%)	30872 (27.8%)
Missing	5066 (3.1%)	721 (1.8%)	262 (1.9%)	4083 (3.7%)

### 3.1 Characteristics of patients with minor children

Among the 39,153 identified patients with children, a total of 82,597 minor children (unique individuals) were found (**Table 1**). Among these patients, three out of five (59%) lived with at least one child. The majority of patients with children were female (60%), while 40% were male, a distribution comparable to that of patients without children under 30 years of age (54% female and 47% male) (**Table 1**). The meadian age of patients with minor children was 38.7 years (min-max 18.0-79.8), compared with 29.7 years (min-max 18.0-105) among patients without children.

The most common diagnoses among patients with minor children were DF40–48 nervous and stress-related conditions (31%) followed by DF30–39 affective disorders (25%). A diagnosis within the schizophrenia spectrum (DF20-29) was present in 9% of patients with minor children compared with 21% of the patients without children.

One third of patients with children received public welfare (31%), compared with 36% of patients without children. More patients with children (55%) lived with a partner than patients without children (28%). A greater proportion of patients with children had completed more than 14 years of education (28%) compared with patients without children (16%).

**Figure 1:**
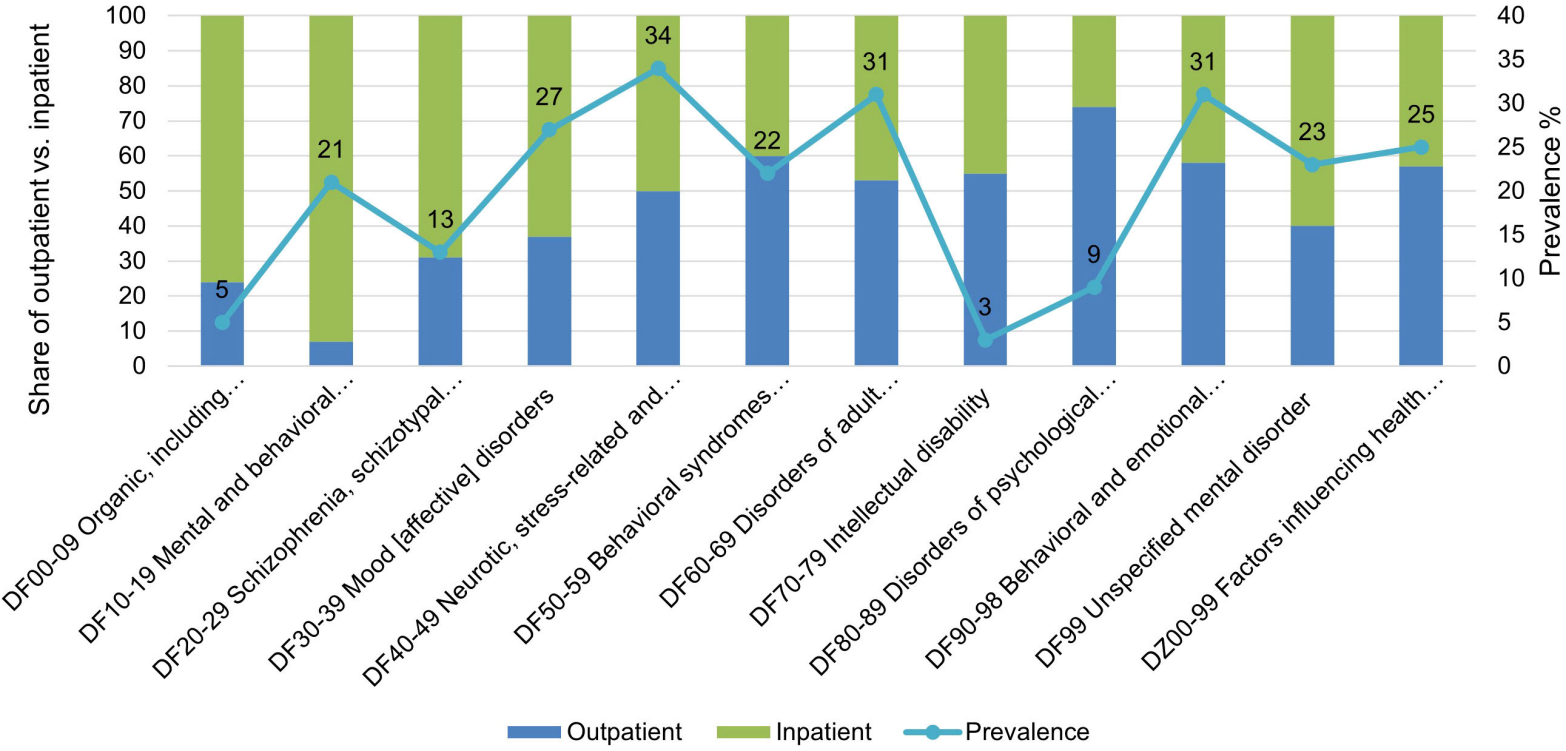
Prevalence of parent patients with minor children.

**Figure 1** shows the prevalence of parenthood among patients across diagnostic categories including the share of at least one inpatient contact versus solely outpatient contacts. The prevalence of parenthood to minor children varied between diagnoses, ranging from 3% among patients with DF7 intellectual disability as the main diagnosis, to 13% in patients with DF2 schizophrenia spectrum disorders, 27% in patients with DF3 affective disorders, and 31% in patients with DF90–98 behavioral and emotional disorders and DF6 personality disorders, and 34% in patients with DF4 neurotic and stress-related disorders.

**Figure 2:**
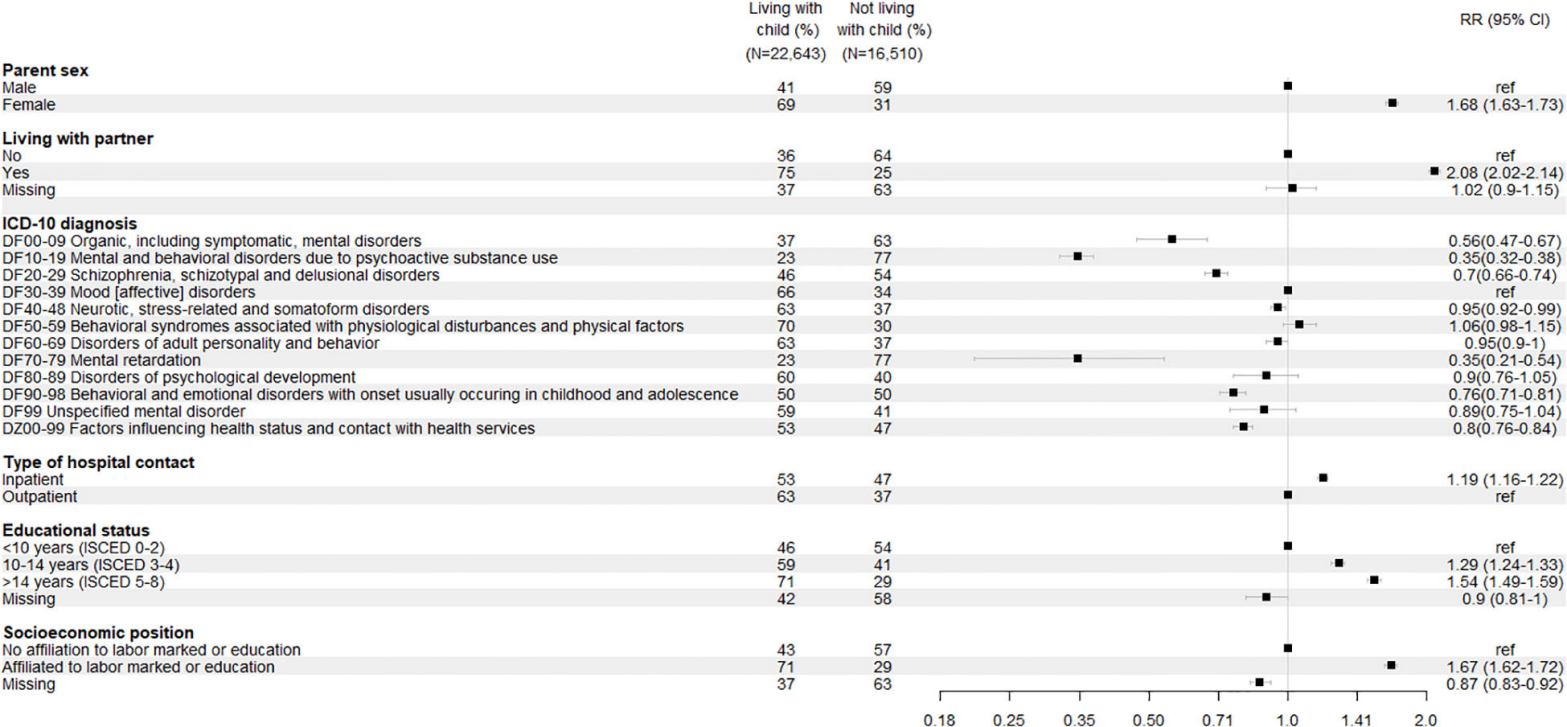
Characteristics of parent patients with minor children.

**Figure 2** summarizes the characteristics of patients who are parents to minor children. Among these patients, females were more likely to live with their child compared with males [RR 1.63 (95% CI 1.63-1.73)]. The parent patients living with a partner were twice as likely to live with their child compared to parent patients not living with a partner (RR 2.02 (95% CI 2.02-2.14). Compared to those with affective disorders (DF3), parent patients diagnosed with DF4, DF5, and DF6 had similar likelihood of living with their child. In contrast, those with DF0, DF1, DF2, DF7, DF9, or DZ were significantly less likely to live with their child (RR range: 0.35-0.89).

A similar pattern was observed regarding educational attainment and self-sufficiency. Parent patients with a long education (ISCED 5-8) or being affiliated to labor market or education were one and a half times more likely to live with their child compared to those with a shorter education (ISCED 0-2) or with no affiliation to the labor market or education.

Approximately half (55%) of the patients who are also parents had been admitted to inpatient psychiatric care during the study period, while the other (45%) half had only outpatient psychiatric contacts (**Table 1**). Parent patients with inpatients contacts were 19% more likely to live with their child compared to those with solely outpatient contacts [RR 1.19 (95% CI 1.16-1.22)].

**Figure 3:**
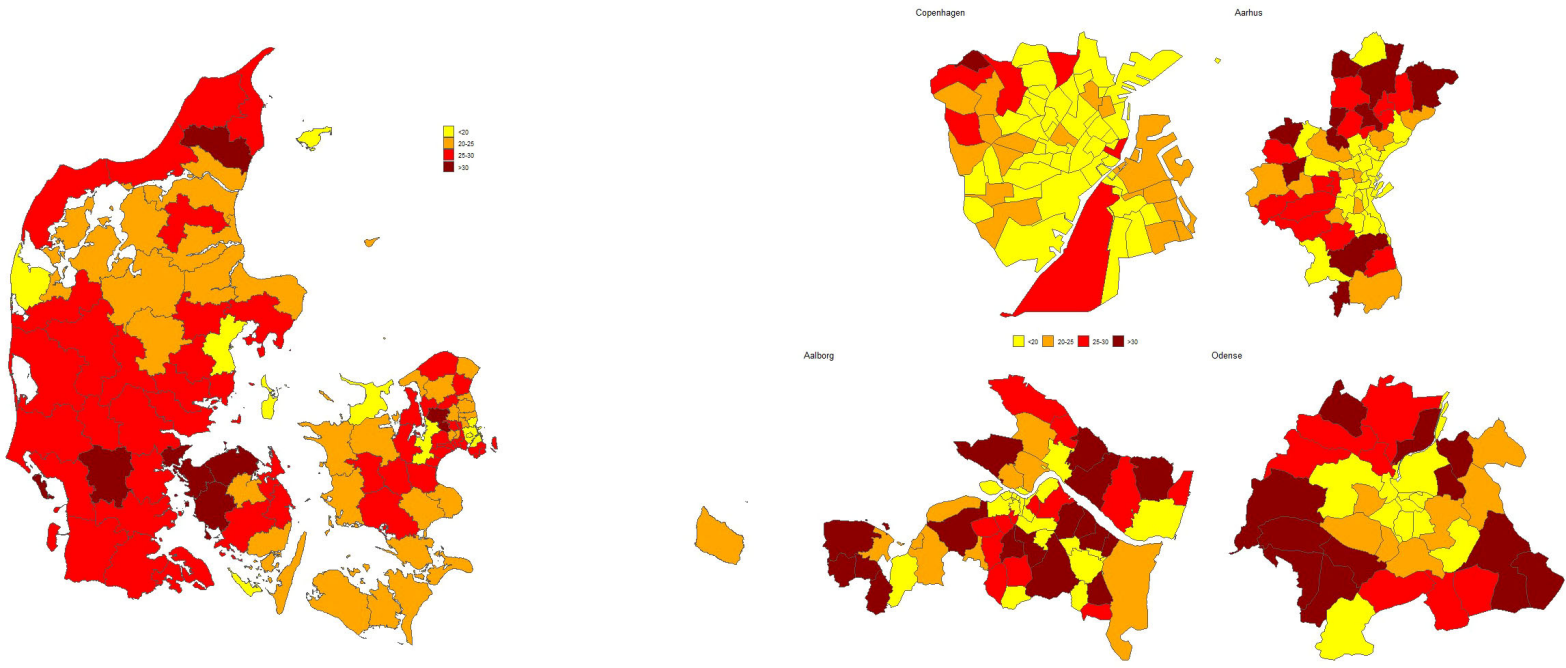
Geographical variation of prevalence of parent patients with minor children.

**Figure 3** illustrates the geographical variation in the prevalence of parenthood among patients in psychiatric hospital services, ranging from below 20% to above 30% across municipalities and within larger cities. Also, variation between the different psychiatric departments ranging from zero to 100%, **Supplementary Figure S1**.

## 4 Discussion

This Danish nationwide study identified that 24.2% of the patients being treated in psychiatric hospitals in Denmark between 2020–2022 had minor children, corresponding to 82,597 unique children below age 18. These findings align with prior research, including Ruud et al. who reported a prevalence of 36% among outpatients (18), and Maybery et al, who found a prevalence of 20.4% in a whole-of-service study (15). Additionally, a recent Czech study reported that 34.6% of inpatients with severe mental illness were parents (16), which is comparable to our findings, considering that two-thirds of the offspring in that study were adults (16). Our study found that 8.5% of patients were parents to solely children aged 18–29 years, leading to an overall prevalence of 32.7% for patients with children aged 0–29 years.

The most common diagnoses among parent patients to minor children were DF40-48 (neurotic, stress-related, and somatoform disorders) (31%) and DF30-39 (affective disorders) (25%), whereas DF2 schizophrenia and other psychotic disorders were seen in only 9% of the parent patients. This contrasts with findings from Howe et al., who reported that 21.5-35.2% of the parents had psychotic disorders (14), and Ruud et al., who reported a prevalence of 13% for schizophrenia and related disorders (18). The variation in diagnostic distribution may stem from differences in psychiatric service structure, treatment approaches, and hospitalization thresholds across countries. Our study’s inclusion of both inpatient and outpatient populations further distinguishes our findings from those in previous research, which often focused on a narrower patient group.

A novel contribution of this study is the identification of substantial geographic variation in the prevalence of parenthood among patient within psychiatric hospital services across Denmark ranging from below 20% to above 30% in different municipalities. This geographic disparity has not previously been studied using a comprehensive nationwide approach but is consistent with earlier research on the prevalence of children with parents with mental disorders in Denmark (10).

In general, parent patients had a higher socioeconomic position (longer educational attainment and self-sufficient/not receiving public benefits) than patients without children. Socioeconomic advantage also characterized the parent patients living with their children compared with parent patients not living with their children. However, it is important to keep in mind that children of parents with mental illness have markedly higher risks of broad socioeconomic adversity than other children (13).

Furthermore, the observed differences in diagnoses and sociodemographic characteristics between patients with and without minor children likely reflect, at least in part, a selection effect. Individuals with greater social and functional stability—such as those in partnerships or with higher education—may be more likely to become parent in the first place. Conversely, patients with conditions such as schizophrenia, intellectual disability, or organic mental disorders may be less likely to have children due to both biological and social factors. This has been demonstrated in prior research showing markedly lower fertility rates among individuals with a range of mental disorders (32). This selective parenthood must be considered when interpreting our findings, as some differences may stem from this initial selection rather from the impacts of parenthood itself.

The relative risks presented in this are based on unadjusted comparisons. Our primary aim was descriptive, focusing on mapping the prevalence and characteristics of parents in psychiatric hospital services. The unadjusted estimates should therefore be interpreted as indicative patterns within the population rather than causal effects.

### 4.1 Strengths

This study has several strengths. By using the national Danish registers, which are considered valid regarding accuracy and completeness (33), we were able to include all patients with at least one contact with the public psychiatric hospitals in Denmark in 2020-2022. To our knowledge, this is the first nationwide study to systematically assess parenthood among patients in psychiatric hospital services in Denmark. Additionally, the study included both inpatient and outpatient populations, capturing a more complete picture, inclusive diagnoses, and sociodemographic characteristics, of parental prevalence being treated in psychiatric hospitals, which are absent or underreported in other studies (16–18).

### 4.2 Limitations

Despite these strengths, some limitations must be acknowledged. The study only identified parenthood among patients who sought mental health care within the hospital system, thereby excluding parents with mental illness who did not receive hospital-based treatment during the study period, but who may have received treatment from, for example, private practitioning psychiatrists. Additionally, we were unable to include data on parents receiving addiction treatment through municipal services.

In this study, patients with more than one diagnosis were assigned a primary diagnosis based on the highest-ranked diagnosis according to the ICD-10 hierarchy. While this approach is commonly used in register-based studies to ensure consistency and comparability across large populations, it may not fully reflect the complexity of clinical presentations or comorbidities. In some cases, a less severe but more functionally impairing disorder may be more relevant to a patient’s parental role. As such, this method could potentially underrepresent the prevalence of parenthood within diagnostic categories associated with milder disorders, and should be interpreted with this limitation in mind.

Additionally, variable such as co-residence with children and socioeconomic position were measured at the time of the patient’s first contact with psychiatric hospital services during the study period. These characteristics may change over time, an in particular, the onset or progression of severe mental illness may lead to disruptions in family structure and employment status. As such, our findings should be interpreted in the light of the fact that these variables reflect the situation at first contact and may not represent long-term or post-diagnosis trajectories.

### 4.3 Implications

The high prevalence of patients in the psychiatric hospital system who are also parents calls for attention from both policy makers and health professionals. Given the well-documented adverse consequences of parental mental illness on children (1–7), it is critical to implement structured screening and support strategies within psychiatric services. A recent Danish report highlights that overcoming identified barriers requires structured action-oriented guidelines, sustained leadership attention, and practical tolls such as reminders and visual patient information (34). The significant variation in prevalence across diagnostic groups and geographic regions suggests that tailored, location-specific interventions may be necessary to effectively reach and support these families.

Ensuring that children of patients within psychiatric hospital services receive adequate support requires clear guidelines and policies. While countries such as Norway and Sweden have legal frameworks mandating the identification of children of patients being treated in psychiatric hospitals, Denmark lacks a similar systematic approach. The Danish Child’s Law obligates professionals to report concerns regarding children’s well-being, but specific guidelines for proactively identifying and supporting these children remain inconsistent. This study underscores the need for a national strategy to bridge this gap and provide comprehensive, evidence-based interventions for patients in psychiatric hospital services who are parents, as well as their children.

Further, our findings highlight the influence of social determinants on mental health, reinforcing evidence that structural factors shape the life circumstances of patients with mental disorders. Addressing these social determinants through mental health policies could help break intergenerational cycles of disadvantage and improve outcomes for both patients and their children (9).

## 5 Conclusions

Approximately one in four Danish patients being treated in psychiatric hospitals have minor children. This prevalence varied by diagnosis and geography, underscoring the importance of systematic identification and evidence-based, tailored intervention strategies for these families. Addressing the needs of these families through targeted policies and resource allocation is critical to mitigate the intergenerational impacts of parental mental illness and improve outcomes for both parents and children.

## Data Availability

The data analyzed in this study is subject to the following licenses/restrictions: The data that support the findings of this study is available from the Statistics Denmark, but restrictions apply to the availability of these data, which were used under licence for the current study, and so are not publicly available. Questions or requests concerning these data are directed to the corresponding author Signe Heuckendorff. Requests to access these datasets should be directed to Signe Heuckendorff, s.heuckendorff@rn.dk.

## Supplementary material

Supplementary Figure 1 Variation of prevalence of parent patients in different psychiatric departments.
