# Supplementary figures and images for: Prevalence and sociodemographic characteristics of parents among patients treated in adult psychiatric hospitals – a register-based nationwide study
Source: Front Psychiatry. 2025 Sep 3;16:1654760. doi: 10.3389/fpsyt.2025.1654760 (PMC12440855; doi:10.3389/fpsyt.2025.1654760)

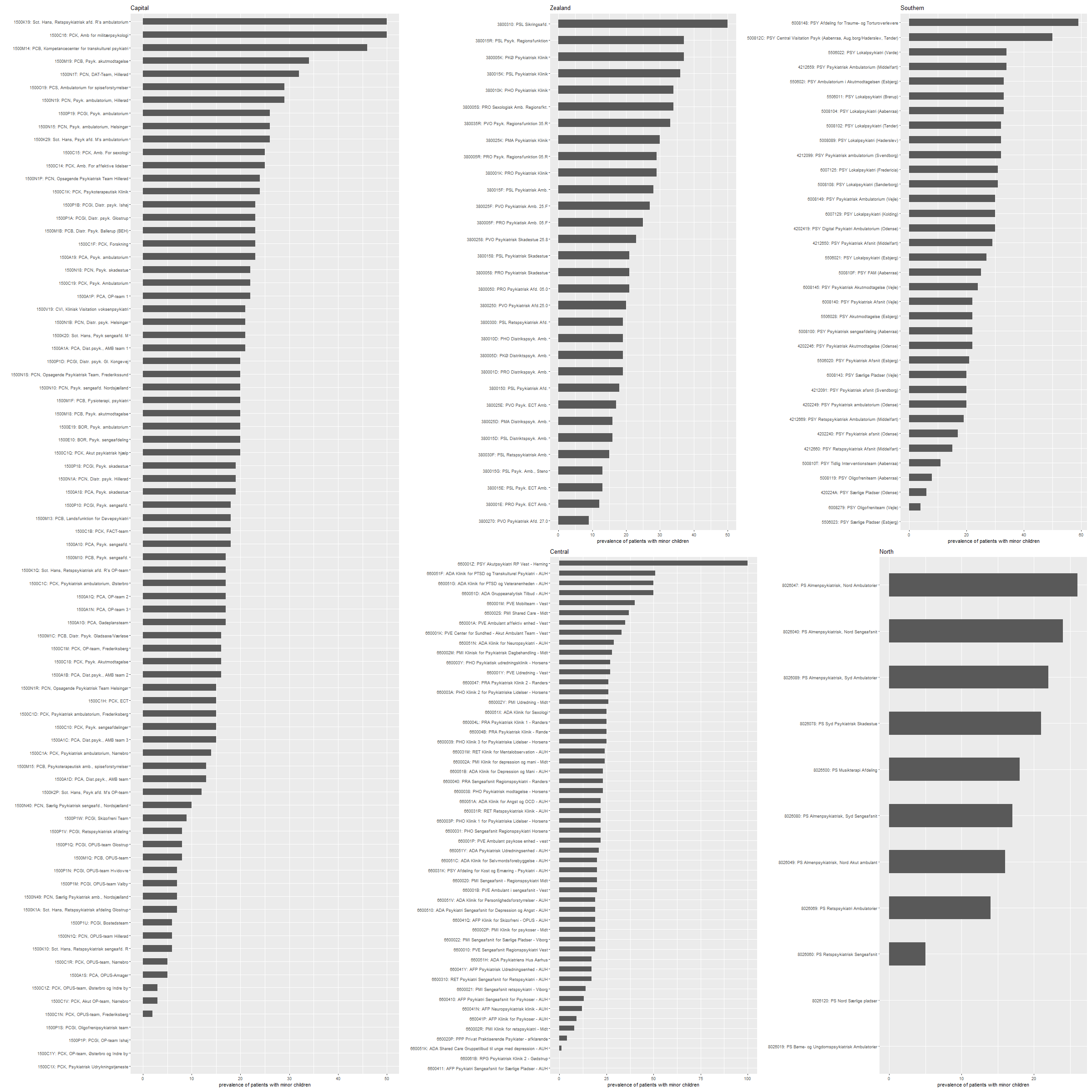

Supplement: Supplementary Figure 1 — Variation of prevalence of parent patients in different psychiatric departments. [file Image1.png]
